# *VRK1* ‐Related Motor Neuropathy With Upper Motor Neuron Signs and Selective Muscle Involvement

**DOI:** 10.1111/jns.70145

**Published:** 2026-07-14

**Authors:** Manoella Guerra de Albuquerque Bueno, Diogo Fernandes dos Santos, Alexander M. Rossor, Matilde Laura, Alejandro Horga, Rodrigo Siqueira Soares Frezatti, Eduardo Vaz de Sousa Ferreira, Julian C. Blake, Jasper M. Morrow, Mary M. Reilly, Wilson Marques Júnior, Pedro José Tomaselli

**Affiliations:** ^1^ Department of Neuroscience and Behavioral Sciences, Ribeirão Preto Medical School University of São Paulo Ribeirão Preto São Paulo Brazil; ^2^ Clinical Hospital Federal University of Uberlândia (HC/UFU) Uberlândia Brazil; ^3^ MRC Centre for Neuromuscular Diseases National Hospital for Neurology and Neurosurgery and UCL Institute of Neurology, Queen Square London UK; ^4^ Department of Clinical Neurophysiology Norfolk and Norwich University Hospital Norwich UK

**Keywords:** distal hereditary motor neuropathy, juvenile ALS, motor neuron disease, muscle MRI, upper motor neuron signs, VRK1

## Abstract

**Introduction:**

Hereditary motor neuropathies (HMN) represent a heterogeneous group of disorders with wide clinical and genetic variability. Despite advances in molecular diagnostics, approximately 50% of cases remain genetically unresolved, particularly those where distinguishing length‐dependent motor neuropathy from motor neuron disorder with disproportionate segmental involvement is a challenge. Variants in the VRK1 gene, originally described in association with pontocerebellar hypoplasia, are now known to produce a broad clinical spectrum, including amyotrophic lateral sclerosis, dHMN, and less frequently, spastic paraplegia.

**Objectives:**

This study's aim was to characterize the clinical presentation, electrophysiological findings, and muscle MRI patterns associated with VRK1‐related motor neuron disease in a cohort of nine patients from five unrelated families.

**Methods:**

Five unrelated families with inherited motor neuropathy were investigated using next‐generation sequencing techniques, including targeted gene panels or whole‐exome sequencing, with subsequent confirmation by Sanger sequencing. A total of nine affected individuals underwent detailed clinical evaluation, nerve conduction studies (NCS), electromyography (EMG), and whole‐body muscle MRI (wbMRI).

**Results:**

Nine affected individuals carrying biallelic VRK1 variants were evaluated. In most cases (66%), symptom onset occurred during the first decade of life. All patients presented with gradually progressive distal muscle weakness. Mean Medical Research Council (MRC) scores were 2.8 for ankle dorsiflexion and 1.9 for plantar flexion. Sensory nerve conduction studies were normal in all individuals evaluated (8/9), although mild sensory complaints were reported in four patients. Muscle cramps were observed in two‐thirds of the cohort, while fasciculations were uncommon (11%). EMG findings consistently demonstrated a neurogenic pattern with predominant distal involvement, and evidence of both acute and chronic denervation was present in four patients. Whole‐body muscle MRI, available for all patients, revealed a consistent pattern of fatty infiltration predominantly affecting posterior muscle compartments, with minimal STIR signal changes. Brain and spinal imaging, performed in all individuals, showed no abnormalities.

**Interpretations:**

Biallelic mutations in VRK1 are associated with a recognizable form of motor neuron disease characterized by features of dHMN combined with upper motor neuron involvement, along with a distinctive posterior‐predominant pattern on muscle MRI. Identifying this phenotype, a known presentation of VRK1‐related disorders, highlights the importance of targeted genetic testing in unresolved cases of hereditary motor neuropathy.

## 1 Introduction

Hereditary motor neuropathies (HMNs) are a genetically and clinically heterogeneous group of disorders characterized by progressive motor weakness with minimal or no sensory involvement [1, 2]. An increasing number of genes have been identified, collectively accounting for nearly half of all cases [3]. Despite the widespread use of next‐generation sequencing (NGS), most patients remain without a molecular diagnosis [3]. This diagnostic gap is particularly evident given the challenges in distinguishing length‐dependent motor neuropathy from motor neuron disorder with disproportionate segmental involvement. The association between motor neuropathy and pyramidal features has been recognized since the early descriptions of “peroneal muscular atrophy with brisk reflexes” [4] and was later delineated as a distinct clinical entity by Dyck and Lambert [5]. Early reports described autosomal dominant inheritance; however, subsequent identification of families with autosomal recessive transmission supported the concept that this group of disorders is genetically heterogeneous [6]. More recently, genes traditionally associated with neurodevelopmental disorders have been recognized to present with combined upper and lower motor neuron (UMN/LMN) involvement [7].

Among these, VRK1 (vaccinia‐related kinase 1) has emerged as a gene with marked phenotypic variability. Initially associated with spinal muscular atrophy (SMA) with pontocerebellar hypoplasia type 1A [8], biallelic pathogenic VRK1 variants are now recognized across a broad clinical spectrum, ranging from juvenile or early‐onset amyotrophic lateral sclerosis (ALS) to distal hereditary motor neuropathy (dHMN; also referred to as distal spinal muscular atrophy, dSMA), with variable cerebellar, cognitive, and other neurodevelopmental manifestations [8, 9, 10, 11]. A recent systematic review of 53 individuals with pathogenic VRK1 variants identified ALS as the most frequent clinical presentation, followed by dHMN, SMA with pontocerebellar hypoplasia type 1, hereditary motor and sensory neuropathy, primary microcephaly, and spastic paraplegia, highlighting frequent UMN involvement in VRK1‐associated disease [12]. Collectively, these findings suggest that *VRK1*‐associated disease represents a multisystem disorder rather than a condition confined to the motor neuron, with variable involvement of the central and peripheral nervous systems. This expanding clinical spectrum parallels that observed in other genes associated with motor neuron disorders, including *SIGMAR1* and *SETX*, in which pathogenic variants produce a continuum of phenotypes with variable upper and lower motor neuron involvement [13, 14].

In this study, we report detailed clinical and electrophysiological assessments, together with whole‐body muscle MRI (wbMRI), to characterize the pattern and extent of muscle involvement in a cohort of patients with confirmed biallelic pathogenic VRK1 variants.

## 2 Methods

### 2.1 Study Design and Participants

Nine patients from five families were investigated as part of a larger study aimed at establishing the molecular diagnosis in individuals with suspected inherited neuropathies using NGS approaches. One patient was evaluated at the National Hospital for Neurology and Neurosurgery, Queen Square, London, UK; two siblings were assessed at the Federal University of Uberlândia, Brazil; and the remaining patients were followed at the Clinical Hospital of Ribeirão Preto, Brazil. The study was approved by the National Hospital for Neurology and Neurosurgery Research Ethics Committee/Central London REC 3 (09/H0716/61) and by the local ethics committee in Brazil (CAAE: 33838220.9.0000.5440). Written informed consent was obtained from all participants or their legal guardians in accordance with the Declaration of Helsinki.

### 2.2 Clinical Assessment and Ancillary Investigations

Clinical data were collected during in‐person visits using a standardized form with predefined variables, including cerebellar and sensory assessment, evaluation for fasciculations, systematic examination of upper motor neuron (UMN) signs, and detailed characterization of weakness distribution. Nerve conduction studies (NCS) were available for eight out of nine patients and were retrieved from their medical records for analysis. Whole‐body muscle MRI (wbMRI) was performed in eight patients. T1‐weighted and short tau inversion recovery (STIR) sequences were acquired. Muscle groups evaluated are detailed in Table S1. Muscle fatty infiltration and oedema were assessed semiquantitatively by a single reader (R.F.). T1‐weighted images were scored using the modified 5‐point Mercuri scale [15]: 0 = normal; 1 = mild fatty streaks; 2 = < 30% fatty infiltration; 3 = 30%–60%; 4 = > 60%; 5 = complete fat replacement. STIR images were rated using a 3‐point scale [16]: 0 = none; 1 = mild hyperintensity; 2 = marked hyperintensity. Each muscle was categorized across the cohort as: Spared (S): no patient with Mercuri ≥ 2; Variable (V): some patients affected or Involved (I): all patients with Mercuri ≥ 2.

### 2.3 Genetic Analysis

NGS was performed in all probands. Whole‐exome sequencing (WES) was conducted in cases 1, 2, and 6, while cases 4 and 5 underwent targeted gene panel analysis (Table S2). Segregation analysis and variant confirmation in affected family members were performed by Sanger sequencing. Variants were interpreted and classified according to the American College of Medical Genetics and Genomics (ACMG) guidelines [17].

## 3 Results

### 3.1 Clinical Features

**FIGURE 1:**
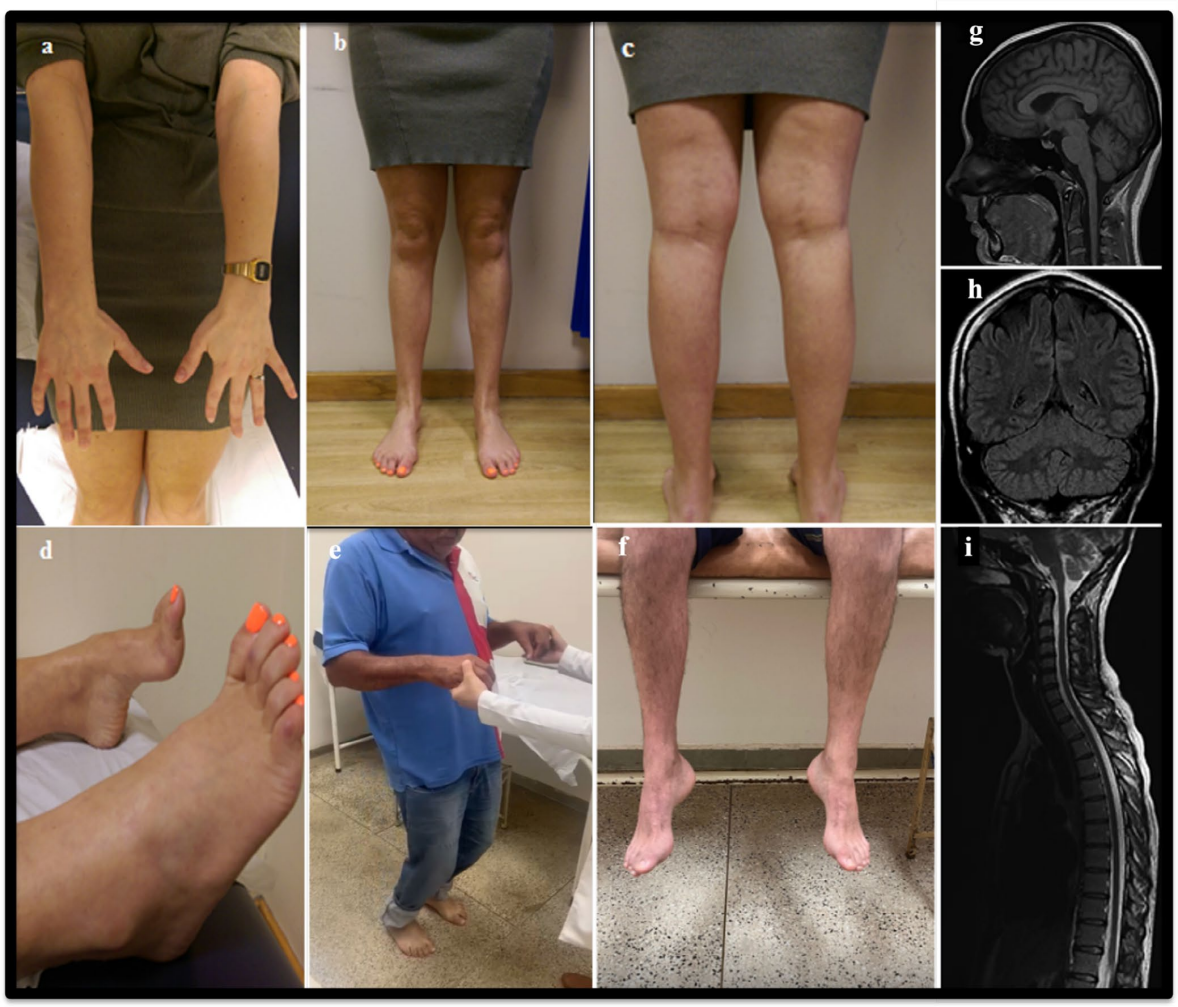
Pictures of the patient I.01 showing pes cavus and wasting distally in the lower limbs with spared upper limbs (a–d). Pictures of patient V.07 showing plantar flexion weakness with knee bending while trying to stand on tip‐toe (e) and wasting distally (f). Sagittal and coronal T1‐weighted MRI showing normal brain. (g, h) Sagittal T2‐weighted MRI showing normal spinal cord (i).

Five males and four females with symptom onset ranging from two to 17 years old were evaluated. Clinical features are summarized in Table 1. All patients achieved normal developmental milestones and had unremarkable early neurodevelopment, with no history of learning difficulties. The initial manifestation in all cases was distal lower limb weakness beginning within the first two decades of life. Patients reported difficulty walking and/or running as the earliest symptom. Motor impairment progressed slowly in all individuals. Muscle cramps were reported in approximately two‐thirds of patients, predominantly affecting the lower limbs. Fasciculations were described by only one patient and were confined to the calves. Mild dysphagia for solid food was present in a single case (case IV.5). None of the patients reported incoordination or dyspnoea. Neurological examination revealed a consistent phenotype across patients, characterized by predominant lower limb weakness with preferential involvement of the posterior compartment of the legs. Accordingly, ankle plantar flexion was more severely affected than dorsiflexion. Upper limb involvement generally occurred later in the disease course. Most patients developed moderate distal weakness in the upper limbs, while proximal muscles remained largely spared (Figure 1). Upper motor neuron signs were present in all patients and included brisk deep tendon reflexes and mildly increased muscle tone in the lower limbs. Coordination was normal in all individuals. Vibration and proprioception were preserved in all cases. However, three siblings from family V exhibited mild pinprick sensory abnormalities (cases V.6, V.7, and V.8).

**TABLE 1:** Clinical, genetic, and neurophysiological features of patients with biallelic *VRK1* variants.

Family	I	II		III	IV	V			
Case	1	2	3	4	5	6	7	8	9
Sex	Female	Male	Female	Female	Male	Female	Male	Male	Male
Country	UK	Brazil	Brazil	Brazil	Brazil	Brazil	Brazil	Brazil	Brazil
Age at Onset (years)	First decade	First decade	First decade	6	2	First decade	15	16	17
Consanguinity	No	No	No	No	Yes	No	No	No	No
Transcript	NM_003384.3	NM_003384.3	NM_003384.3	NM_003384.3	NM_003384.3	NM_003384.3	NM_003384.3	NM_003384.3	NM_003384.3
VRK1 Variant (cDNA)	c.683C>T, c.1072C>T	c.265C>T, c.452G>A	c.265C>T, c.452G>A	c.37_38del, c.1160G>A	c.637 T>C, c.637 T>C	c.452G>A, c.1012A>T	c.452G>A, c.1012A>T	c.452G>A, c.1012A>T	c.452G>A, c.1012A>T
VRK1 Variant (Protein)	p.Thr228Met, p.Arg358*	p.Arg89*; p.Arg151Gln	p.Arg89*; p.Arg151Gln	p.Ser13LeufsTer18; p.Arg387His	p.Tyr213His	p.Arg151Gln, p.Lys338Ter	p.Arg151Gln, p.Lys338Ter	p.Arg151Gln, p.Lys338Ter	p.Arg151Gln, p.Lys338Ter
ACMG classification	3; 5	5; 4	5; 4	4; 5	5	4; 4	4; 4	4; 4	4; 4
PMID	31 090 908;19 646 678	32 242 460; not described	32 242 460; not described	Not described; 31 837 156	32 365 420	Not described; Not described	Not described; Not described	Not described; Not described	Not described; Not described
Initial symptom	Difficulty running	Difficulty running	Difficulty running	Funny gait	Frequent falls	Difficulty running	Distal weakness	Distal weakness	Frequent falls
Distal weakness (UL/LL)	No/yes	No/yes	No/yes	Yes/yes	Yes/yes	Yes/Yes	Yes/Yes	Yes/Yes	Yes/Yes
Proximal Weakness (UL/LL)	No/no	No/no	No/no	No/yes	Yes/yes	Yes/Yes	No/Yes	No/Yes	No/Yes
Muscle Tone (UL/LL)	Normal/normal	Normal/normal	Normal/normal	Normal/slightly increased	Normal/normal	Normal/increased	Normal/increased	Increased/increased	Normal/increased
Deep tendon reflexes (UL/LL)	Brisk/brisk	Brisk/brisk, ankle absent	Brisk/brisk, ankle absent	Brisk/brisk, ankle absent	Brisk/brisk, ankle absent	Very brisk/very brisk, ankle absent	Brisk/Very brisk, ankle absent	Very brisk/very brisk, ankle absent	Brisk/brisk, ankle absent
Plantar response	Down	Down	Down	Mute	Mute	Down	Mute/Up (right/left)	Up	Mute
Cramps	Yes	Yes	Yes	Yes	No	Yes	No	Yes	No
Fasciculations	Yes	No	No	No	No	No	No	No	No
Sensory signs	No/no	No/no	No/no	No/no	No/no	No/Yes	Yes/Yes	Yes/Yes	No/No
Bulbar involvement	No	No	No	No	Yes	No	No	No	No
Ankle dorsiflexion (MRC)	4+	4	4	4−	0	4−	1	2	2
Ankle plantar flexion (MRC)	4	3	4—	2	0	2	0	1	1
First dorsal interosseous (MRC)	—	—	—	3	3	3	3	4	3
Abductor pollicis brevis (MRC)	—	—	—	4	4	4	4	4	4
Brain and spinal cord MRI	Normal	Normal	Normal	Normal	Normal	Normal	Normal	Normal	Normal

*Note:* Clinical and molecular characteristics of nine affected individuals from five unrelated families carrying biallelic *VRK1* variants (transcript reference: NM_003384.3). Age at onset is reported in years or by decade of life. Variants are described at the cDNA and protein levels according to HGVS nomenclature. ACMG classification is shown for each variant (1 = benign; 2 = likely benign; 3 = variant of uncertain significance; 4 = likely pathogenic; 5 = pathogenic). PMIDs refer to previously published reports describing the variant when available; “not described” indicates absence of prior published reports. Muscle strength is graded according to the Medical Research Council (MRC) scale. Deep tendon reflexes are described qualitatively; ankle reflex status is specified when absent. Plantar responses are reported as down‐going (flexor), up‐going (extensor), or mute. Sensory signs are indicated separately for upper and lower limbs. MRI findings refer to brain and spinal cord imaging.

Abbreviations: LL, lower limbs; UL, upper limbs.

### 3.2 Neurophysiological Findings

Sensory nerve action potentials were normal in available tests. Motor nerve conduction studies in patient IV.5 exhibited reduced compound muscle action potential (CMAP) amplitudes in all motor nerves in both upper and lower limbs. All other patients had normal CMAPs. Despite relatively preserved motor conduction parameters, needle electromyography (EMG) revealed a more widespread neurogenic pattern in all patients. Chronic denervation changes were observed diffusely in both upper and lower limbs. Active denervation was limited and not a prominent feature (Table S3).

### 3.3 Genetic Findings

All individuals carried biallelic variants in VRK1 (RefSeq: NM_003384.3), consistent with autosomal recessive inheritance, either in homozygosity or in compound heterozisoty proved after segregation analysis (Table 1). Only one individual (Case IV.5) harbored a homozygous missense variant, c.637T>C (p.Tyr213His), in the setting of reported consanguinity. All remaining cases carried compound heterozygous variant combinations, most commonly consisting of at least one loss‐of‐function (LoF) allele (nonsense or frameshift) in trans with a missense variant (Table 1). Three variants were not previously described (p.Arg151Gln, p.Ser13LeufsTer18, p.Lys338*). Structurally, the four missense variants identified in our cohort cluster into two functional regions. The p.Arg151Gln, p.Thr228Met, and p.Tyr213His are located within the catalytic kinase domain, with p.Tyr213His directly affecting the activation loop and associated with severe enzymatic impairment [18]. In contrast, p.Arg387His resides in the C‐terminal regulatory region and preserves global kinase activity while selectively impairing chromatin interaction and H2AX phosphorylation [19]. The p.Arg151Gln variant is located within the N‐terminal catalytic kinase domain. Substitution of a positively charged arginine by a neutral glutamine residue may disrupt electrostatic interactions essential for structural stability and catalytic efficiency, potentially resulting in partial loss of kinase activity and impaired phosphorylation of nuclear substrates involved in RNA processing and chromatin regulation. It was in trans with two different loss‐of‐function alleles in independent families (p.Arg89* in family II and p.Lys338* in family V), and was observed in six affected individuals overall, supporting a likely pathogenic classification in the context of autosomal recessive VRK1‐related disease. This structural stratification suggests a gradient of functional impairment, potentially contributing to phenotypic variability across individuals.

### 3.4 Whole‐Body Muscle MRI

**FIGURE 2:**
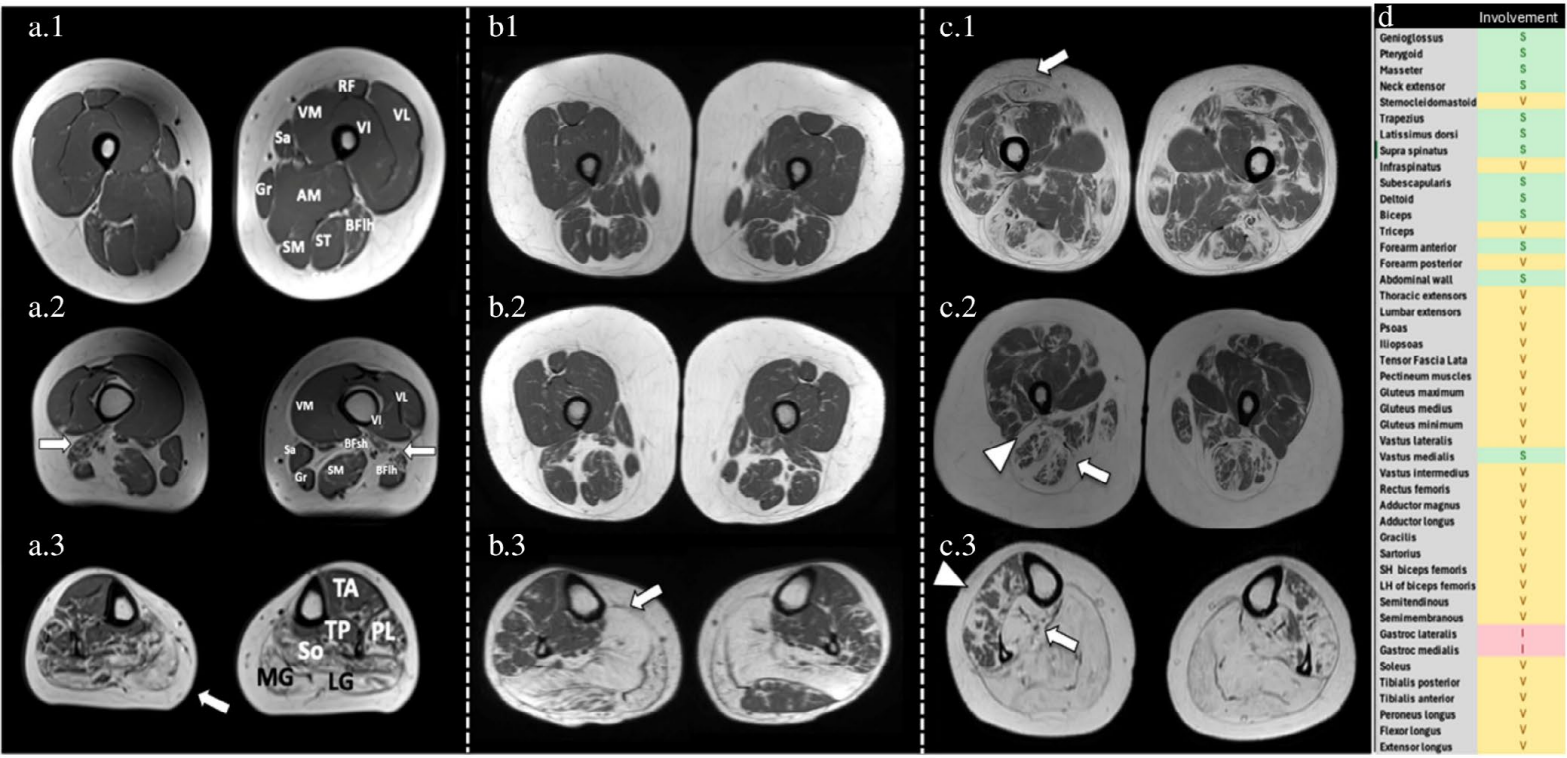
Progression of lower‐limb muscle involvement across disease severity. T1‐weighted axial muscle MRI images from three representative patients with increasing disease severity: Mildly affected (A), moderately affected (B), and severely affected (C). Each column corresponds to a single patient (first: Patient I.1; second: Patient V.6; third: Patient V.8). Upper, middle, and lower rows depict upper thigh, mid‐thigh, and calf levels, respectively. In panel A, anatomical muscle labels are provided on the right. In the calves (A3–C3), early posterior compartment predilection is evident from the mildly affected stage (A3), predominantly involving the gastrocnemius. In the moderate stage (B3), additional involvement of the soleus is observed, with relative preservation of the peroneus longus, tibialis anterior, and extensor hallucis longus. In the severe stage (C3), progression includes involvement of the tibialis posterior—previously spared—as well as the peroneus longus, tibialis anterior, and extensor hallucis longus. Despite advanced fatty infiltration, residual preserved muscle areas (“muscle islands”) remain detectable. At the mid‐thigh level (A2–C2), preferential involvement of the short head of the biceps femoris over the long head is observed in the mild and moderate stages. In the severe stage (C2), the posterior compartment shows marked predominance, with complete fatty infiltration of the short head of the biceps femoris and moderate involvement of the semitendinosus, semimembranosus, long head of the biceps femoris, and adductor magnus. At the upper thigh level (A1–C1), no significant abnormalities are observed in the mild and moderate stages (A1–B1). In the severe stage (C1), involvement of the posterior compartment and predominant fatty infiltration of the rectus femoris within the anterior compartment are evident. (D) A summary heat map displayed on the right indicates the distribution of involved, spared, and variably affected muscles across the entire cohort, highlighting the preferential involvement of posterior compartments in both calves and thighs.

STIR hyperintensities were uncommon and, when present, were mild and confined to distal muscles. In contrast, T1‐weighted imaging demonstrated a clear posterior compartment predominance in both the thighs and legs, consistent with previous reports [12, 20]. Muscle MRI revealed a severity‐dependent pattern of fatty infiltration, with early and preferential involvement of the gastrocnemius and the short head of the biceps femoris. With disease progression, fatty replacement extended to adjacent posterior compartment muscles and, in advanced stages, to selected anterior compartment muscles. Despite advanced fatty infiltration, residual preserved muscle areas (“muscle islands”) remain detectable [21]. Notably, upper thigh muscles remained largely preserved until late in the disease course (Figure 2).

## 4 Discussion

We describe a cohort of patients with biallelic pathogenic variants in VRK1 presenting with a slowly progressive distal motor neuronopathy accompanied by UMN signs. The phenotype was consistent across all individuals and characterized by preferential and more severe involvement of the posterior compartment of the legs, brisk reflexes, mildly increased tone in the lower limbs, without cerebellar involvement.

Clinically, all individuals in this cohort presented with distal lower‐limb weakness beginning within the first two decades of life, consistent with some prior reports [10, 22]. Among our patients, weakness predominantly affected the posterior compartment of the legs, with ankle plantar flexion more severely affected than dorsiflexion. Upper‐limb involvement was generally milder and distal‐predominant. Although some patients reported mild distal sensory complaints affecting nociception, large‐fiber sensory modalities and sensory nerve conduction studies were preserved in all cases. Motor nerve conduction studies were normal in most cases. However, needle electromyography demonstrated a diffuse neurogenic process involving both cervical and lumbosacral segments, with evidence of chronic denervation in all cases and limited active denervation in up to half of them. Despite the presence of combined upper and lower motor neuron features, the pattern of weakness was symmetric and slowly progressive. Although the distribution was clearly nonlength‐dependent, with preferential involvement of the posterior compartment, distal segments were consistently more affected. Disease progression was remarkably slow, and bulbar involvement was observed in only one individual. The split‐hand phenomenon was not identified in any patient in this cohort. The wbMRI provided additional important information. Fatty infiltration predominated in posterior thigh and calf compartments, with relative sparing of anterior muscles early in the disease. STIR hyperintensities were rare and mild, suggesting limited ongoing acute denervation. Similar MRI distributions have been reported in IGHMBP2 [23, 24], SIGMAR1 [13, 14, 25], and SETX [26]. Importantly, these genes lead to motor neuronopathies with broader involvement: IGHMBP2 causes SMARD1, which features early diaphragmatic paralysis and CMT2S, eventually presenting as late onset [27]. SIGMAR1 and SETX mutations are linked to pyramidal signs and upper motor neuron features, going beyond simple axonopathy [14]. Such posterior predominance parallels what is seen in 5q SMA, where typically gluteus muscle is worse than iliopsoas; triceps worse than biceps and hamstrings worse than quadriceps involvement are consistently described [15, 28]. In contrast, sporadic ALS typically presents with a proximal‐to‐distal gradient rather than a posterior to anterior one. In opposition to our findings, frequent and prominent STIR hyperintensities in paraspinals, psoas, gluteus medius, and tongue are well described in ALS patients [29, 30]. These findings are consistent with ongoing denervation [29, 31] and were not observed in our cases. Supporting that our patients show a consistent slowly progressive hereditary motor neuronopathy rather than early onset ALS. Additionally, although classified as distal hereditary motor neuropathy (dHMN), these disorders do not follow a purely length‐dependent pattern of degeneration; rather, they represent motor neuronopathies, presumably with selective vulnerability of motor neuron subpopulations contributing to the pattern of fatty infiltration [12]. This distinction is clinically suggested and further supported by muscle wbMRI findings, in which a selective pattern of fatty infiltration (predominantly involving posterior leg and thigh muscles) with minimal STIR changes is consistent with a hereditary lower motor neuronopathy, as opposed to sporadic ALS, which is typically characterized by less fatty infiltration relative to atrophy and marked STIR hyperintensity, reflecting more rapid progression. Although ALS was previously described as the most common presentation of VRK1 related neuropathy [12], we did not find any patients with this phenotype, which may be related to the size of the cohort, unknown modifier genes or variant‐specific genotype–phenotype correlations. Furthermore, while the p.Arg321Cys variant has been previously described as ALS‐associated, the clinical phenotype reported is more compatible with a symmetric distal motor neuropathy marked by slow progression and hyperreflexia, without clear evidence of upper motor neuron involvement beyond lower motor neuron dysfunction [29]. Bulbar symptoms were not documented, and electromyography demonstrated only chronic denervation changes [29]. Taken together, these findings suggest that the phenotype reported by Oliveira et al. closely resembles that observed in our cohort.

Motor neuron degeneration in VRK1‐related disorders is thought to result from reduced intracellular protein levels or impaired kinase activity. VRK1 is a ubiquitously expressed nuclear serine/threonine kinase with multiple substrates and functions, including regulation of Cajal body dynamics and RNA metabolism, chromatin remodeling, DNA damage response, and neuronal migration [19, 30]. Importantly, VRK1 is expressed in the spinal cord throughout development and adulthood [32], supporting its role in both neurodevelopmental processes and motor neuron maintenance. Beyond its kinase‐dependent functions, VRK1 also interacts with amyloid precursor protein (APP) to regulate neuronal progenitor migration, partially independent of catalytic activity [32]. Additionally, certain truncating alleles have been implicated in the disruption of the reelin signaling pathway, a key regulator of neuronal positioning [33, 34, 35]. Together, these findings support the concept that disturbances in programs governing neuronal placement, differentiation, and nuclear RNA processing may converge to produce both neurodevelopmental and motor neuron phenotypes.

Importantly, VRK1 is haplosufficient, as heterozygous carriers are typically asymptomatic, and clinical manifestations require biallelic pathogenic variants. Accordingly, the phenotypic variability observed in our cohort likely reflects differential functional impairment imposed by specific variants on residual VRK1 activity. Notably, the only patient presenting with bulbar involvement and marked axonal loss, evidenced by diffusely reduced CMAP amplitudes, harbored the p.Tyr213His variant in homozygosity. This variant disrupts the kinase activation loop, leading to a profound reduction in enzymatic activity and disassembly of Cajal bodies, structures essential for RNA processing and splicing [18]. Different from patients harboring homozygous p.Arg358* that behaves like a developmental nuclear loss‐of‐function (dose/localization collapse) producing PCH, whereas p.Tyr213His behaves like a nuclear catalytic hypomorph that preserves early brain morphogenesis but drives later motor neuron degeneration.

In contrast, the other two missense variants (p.Arg151Gln and p.Thr228Met), both located within the catalytic domain but outside the core activation loop, are predicted to alter kinase conformation and reduce catalytic efficiency without causing complete loss of function. Notably, these variants were identified in trans with a null allele, suggesting that the resulting biallelic combination leads to partial rather than total functional impairment. This residual VRK1 activity may be sufficient to preserve early neurodevelopment and maintain relative bulbar function, while still disrupting RNA processing and chromatin regulation in selectively vulnerable motor neurons, thereby accounting for the slower disease progression observed in these patients. This structural–functional gradient supports a genotype–phenotype continuum in VRK1‐related motor neuron disease, whereby variants directly destabilizing the activation loop are associated with more severe phenotypes, whereas variants producing partial catalytic or regulatory impairment result in a more indolent syndrome.

In summary, pathogenic VRK1 variants cause a recurrent motor neuronopathy characterized by early distal lower‐limb weakness, UMN signs, motor‐predominant neurophysiology, and a distinctive posterior‐compartment pattern on muscle MRI.

## Funding

This work was supported by the International Centre for Genomic Medicine in Neuromuscular Diseases (ICGNMD) (MR/S005021/1), the Programa Nacional de Apoio à Atenção da Saúde da Pessoa com Deficiência (PRONAS) (25000.160.096/2014–07), the National Institute of Sciences and Technology (INCT)—Translational Medicine (465 458/20114–9), the Conselho Nacional de Pesquisa (CNPq) (310378/2021‐4), and the Biogen (FE‐CLI‐24‐0005‐05).

## Supporting information


**Table S1:** Muscles evaluated by wb‐MRI divided by segment.
**Table S2:** Genes included in distal hereditary motor neuropathy target panel for case 04 and 05.
**Table S3:** Nerve conduction studies and electromyography of patients 01 to 08. Amp = amplitude, CV = nerve conduction velocity, DML = distal minimal latency, mV = millivolts, m/s = meter/s, ms = milliseconds, μV = microvolts, *P* = proximal, D = distal, NE = not evaluated, Fibs = fibrillations, PSW = positive sharp waves, Ref = reference.

## Data Availability

The data that support the findings of this study are available on request from the corresponding author. The data are not publicly available due to privacy or ethical restrictions.
