# Supplementary material for: VRK1 ‐Related Motor Neuropathy With Upper Motor Neuron Signs and Selective Muscle Involvement
Source: J Peripher Nerv Syst. 2026 Jul 14;31(3):e70145. doi: 10.1111/jns.70145 (PMC13369780; doi:10.1111/jns.70145)
Supplement: Supplementary file 1 — Table S1: Muscles evaluated by wb‐MRI divided by segment. Table S2: Genes included in distal hereditary motor neuropathy target panel for case 04 and 05. Table S3: Nerve conduction studies and electromyography of patients 01 to 08. Amp = amplitude, CV = nerve conduction velocity, DML = distal minimal latency, mV = millivolts, m/s = meter/s, ms = milliseconds, μV = microvolts, P = proximal, D = distal, NE = not evaluated, Fibs = fibrillations, PSW = positive sharp waves, Ref = reference. [file JNS-31-0-s001.docx]

**Supplementary material:**

**Supplementary table 01.** Muscles evaluated by wb-MRI divided by segment.

| **Head and neck** | **Upper limb and Axial** | **Lower Limbs** |
| --- | --- | --- |
| Genioglossus | Supraspinatus | Psoas |
| Medial pterygoids | Infraspinatus | Tensor fasciae latae |
| Lateral pterygoids | Subscapularis | Pectineus |
| Masseter | Deltoid | Gluteus maximus/medius/minimus |
| Neck extensors (grouped) | Biceps brachii | Quadriceps (vastus lateralis/medialis/intermedius, rectus femoris) |
| Sternocleidomastoid | Triceps | Adductors |
| Trapezius | Forearm flexors (grouped) | Gracilis |
|  | Forearm extensors (grouped) | Sartorius |
|  | Abdominal wall | Hamstrings |
|  | Thoracic and lumbar extensors | Gastrocnemius medialis/lateralis |
|  |  | Soleus |
|  |  | Tibialis anterior/posterior |
|  |  | Peroneus longus |
|  |  | Flexor longus |
|  |  | Extensor hallucis longus |

**Supplementary table 02.** Genes included in distal hereditary motor neuropathy target panel for case 04 and 05.

| **Gene name** | **Gene symbol** | **Inheritance pattern** | **Phenotype** |
| --- | --- | --- | --- |
| Aladin | AAAS | AR | Alacrima-achalasia-adrenal insufficiency neurologic disorder |
| Bicaudal D homolog of drosophila 2 | BICD2 | AD | SMALED |
| BSCL2 | BSCL2 | AD | dHMN V, Silver Syndrome |
| Dynactin 1 | DCTN1 | AD | dHMN VIIB |
| Dynein cytoplasmic 1 heavy chain 1 | DYNC1H1 | AD | CMT2, SMALED |
| Fibulin 5 | FBLN5 | AD | CMT1 |
| F-Box Only Protein 38 | FBXO38 | AD | dHMN VI |
| Glycyl-tRNA synthetase 1 | GARS1 | AD | CMT2D, dHMN V |
| Glycogen Branching Enzyme | GBE1 | AR | Glycogen storage disease IV |
| Histidine Triad Nucleotide-Binding Protein 1 | HINT1 | AR | Neuromyotonia and axonal neuropathy |
| Heat-shock 27-KD protein 1 | HSPB1 | AD | CMT2F, dHMN IIB |
| Heat-shock 27-KD protein 3 | HSPB3 | AD | dHMN IIC |
| Heat-shock 27-KD protein 8 | HSPB8 | AD | CMT2L, dHMN IIA |
| Immunoglobulin MU-binding protein 2 | IGHMBP2 | AR | CMT2S, dHMN VI |
| Kinesin Family Member 1A | KIF1A | AD, AR | SPG |
| LAS1-like Ribosome Biogenesis Factor | LAS1L | XLR | Wilson-Turner syndrome |
| Myosin Heavy Chain 14, Nonmuscle | MYH14 | AD | Peripheral neuropathy, myopathy, hoarseness, and hearing loss |
| Pleckstrin Homology Domain - and RhoGEF Domain - Containing Protein G5 | PLEKHG5 | AR | dHMN AR 4, CMT AR |
| Receptor Expression-Enhancing Protein 1 | REEP1 | AD, AR | dHMN AD 12, dHMN AR 6, SPG |
| Senataxin | SETX | AD | ALS 4 |
| Sigma Nonopioid Intracellular Receptor 1 | SIGMAR1 | AR | ALS 16, dMN AR 2 |
| Solute carrier family 52  (Riboflavin transporter), member 1 | SLC52A1 | AR | MADRAS |
| Solute carrier family 52  (Riboflavin transporter), member 2 | SLC52A2 | AR | Brown-Vialetto-Van syndrome 2 |
| Solute carrier family 52  (Riboflavin transporter), member 3 | SLC52A3 | AR | Brown-Vialetto-Van syndrome 1, Fazio-Londe disease |
| Solute Carrier Family 5 (Choline Transporter), Member 7 | SLC5A7 | AD, AR | Myasthenic syndrome, dHMN AD 7 |
| Transient receptor potential cation channel, subfamily V, Member 4 | TRPV4 | AD | dHMN IIC |
| UBIQUILIN 1 | UBQLN1 |  |  |
| VRK SERINE/THREONINE KINASE 1 | VRK1 | AR | dHMN AR 10, PCH1A |

**Supplementary Table 03.** Nerve conduction studies and electromyography of patients 01 to 08. Amp = amplitude, CV = nerve conduction velocity, DML = distal minimal latency, mV = millivolts, m/s = meter/second, ms = milliseconds, µV = microvolts, P = proximal, D = distal, NE = not evaluated, Fibs = fibrillations, PSW = positive sharp waves, Ref = reference.

| **Family** | | 1 | 2 | | 3 | 4 | 5 | | |
| --- | --- | --- | --- | --- | --- | --- | --- | --- | --- |
| **Case** | | 1 | 2 | 3 | 4 | 5 | 6 | 7 | 8 |
| **Sensory Conduction** | **Median Amp (µV) (Ref > 10)** | 17 | 50 | 82 | 17.1 | 12.1 | 37.9 | 12.7 | 21.4 |
|  | **Median CV (m/s) (Ref > 50)** | 52 | 56 | 59 | 67 | 50.7 | 65.3 | 65.7 | 67 |
|  | **Ulnar Amp (µV) (Ref > 10)** | 25 | 54 | 41 | 13.1 | 11.9 | 17.6 | 11.7 | 11.8 |
|  | **Ulnar CV (m/s)**  **(Ref > 50)** | 53 | 59 | 63 | 67.1 | 51.1 | 64.1 | 67.4 | 66.3 |
|  | **Radial Amp (µV) (Ref > 15)** | 55 | 56 | 84 | 35 | 26.3 | 48.6 | 25.9 | 34.6 |
|  | **Radial CV (m/s)**  **(Ref > 50)** | 65 | 63 | 62 | 58.4 | 52.3 | 65.6 | 65.8 | 66.3 |
|  | **Superficial Peroneal Amp (µV)**  **(Ref > 6)** | Not evaluated | 21 | 36 | 27 | 10.7 | NE | NE | NE |
|  | **Superficial Peroneal CV (m/s)**  **(Ref > 40)** | Not evaluated | 42 | 44 | 55 | 44.9 | NE | NE | NE |
|  | **Sural Amp (µV)**  **(Ref > 6)** | 22 | 53 | 41 | 13 | 24.1 | 15.7 | 11.8 | 18.6 |
|  | **Sural CV (m/s)**  **(Ref > 40)** | 45 | 48 | 43 | 58.4 | 47.6 | 52.2 | 46.4 | 50 |
|  |  |  |  |  |  |  |  |  |  |
| **Motor Conduction** | **Median Amp (mV)**  **(Ref > 3,8)** | 10 | 9.3 | 8.6 | 15.8 | 2.9 | 16.3 | 8.24 | 5.87 |
|  | **Median CV (m/s)**  **(Ref > 50)** | 63 | 59 | 58 | 61.4 | 51.6 | 60.3 | 61.7 | 60 |
|  | **Median DML (ms)**  **(Ref > 3,7)** | 3.8 | 3.1 | 2.5 | 3.5 | 5.65 | 2.45 | 3.25 | 3.3 |
|  | **Ulnar Amp (mV)**  **(Ref > 3,8)** | 14.6 | 10.8 | 10.9 | 8.5 | 0.2 | 09.03 | 7 | 7.1 |
|  | **Ulnar CV (m/s)**  **(Ref > 50)** | 61 | 62 | 63 | 63.4 | 42.5 | 71.2 | 64.8 | 64 |
|  | **Ulnar DML (ms)**  **(Ref > 3,7)** | 3.6 | 2.5 | 2.2 | 2.3 | 5.85 | 2.2 | 2.55 | 2.6 |
|  | **Common Peroneal Amp (mV)**  **(Ref > 2,8)** | 6.1 | 8.7 | 3.8 | 6.4 | 0.27 | 3.19 | 7.41 | 5.84 |
|  | **Common Peroneal CV (m/s)**  **(Ref > 40)** | 57 | 49 | 48 | 52.9 | 41 | 52.1 | 50.9 | 50.5 |
|  | **Common Peroneal DML (ms)**  **(Ref > 4)** | 5.4 | 4.4 | 3.5 | 3.8 | 6.4 | 3.75 | 3.6 | 4.75 |
|  | **Posterior Tibial Amp (mV)**  **(Ref > 3,6)** | 8.3 | 7.8 | 10.5 | 6 | NE | NE | 3.74 | NE |
|  | **Posterior Tibial CV (m/s)**  **(Ref > 40)** | 49 | 46 | 51 | 54 | NE | NE | 51 | NE |
|  | **Posterior Tibial DML (ms)**  **(Ref > 4)** | 4.4 | 3.7 | 3.3 | 3.3 | NE | NE | 4.4 | NE |
| **Needle** | **Neurogenic Pattern** | Yes | NE | NE | Yes | Yes | Yes | Yes | Yes |
|  | **Upper Limbs involvement (P/D)** | No/No | NE | NE | No/Yes | Yes/Yes | No/Yes | No/Yes | No/Yes |
|  | **Lower Limbs involvement (P/D)** | Yes/Yes | NE | NE | Yes/Yes | Yes/Yes | No/Yes | Yes/Yes | Yes/Yes |
|  | **Chronic denervation** | Yes | NE | NE | Yes | Yes | Yes | Yes | Yes |
|  | **Spontaneous Activity** | Fibs/PSW | NE | NE | No | Fibs/PSW | No | Fibs/PSW | Fibs/PSW |
